# Problematic Internet Use in University Students Attending Three Superior Graduate Schools in Italy: Is Autism Spectrum Related to Suicide Risk?

**DOI:** 10.3390/ijerph16071098

**Published:** 2019-03-27

**Authors:** Liliana Dell’Osso, Carlo Antonio Bertelloni, Marco Di Paolo, Maria Teresa Avella, Barbara Carpita, Federica Gori, Maurizio Pompili, Claudia Carmassi

**Affiliations:** 1 Department of Clinical and Experimental Medicine, University of Pisa, 56126 Pisa, Italy; liliana.dellosso@med.unipi.it (L.D.); mariateresa91.avella@gmail.com (M.T.A.); juanpagn@yahoo.it (B.C.); claudia.carmassi@unipi.it (C.C.); 2 Department of Clinical, Medical, Molecular Pathology and Critical Medicine, University of Pisa, 56126 Pisa, Italy; marco.dipaolo@unipi.it (M.D.P.); f.gori@ao-pisa.toscana.it (F.G.); 3 Department of Neurosciences, Mental Health and Sensory Organs, Suicide Prevention Center, Sant’Andrea Hospital, Sapienza University, 00189 Rome, Italy; maurizio.pompili@uniroma1.it

**Keywords:** PIU, PUI, internet, autism, AdAS, suicide risk, suicide

## Abstract

*Background*: Over the past decades, problematic internet use (PIU) has dramatically increased, especially among young people. PIU has been recently associated with autism spectrum disorder (ASD) and autistic traits. Subjects with PIU report an increased suicidal risk and the same has been demonstrated among patients with ASD. The aim of this study was to investigate putative PIU rates among students and explore the correlation between autistic traits and suicide risk. *Methods*: A sample of 178 high achieving university students was assessed by means of the Adult Autism Subthreshold Spectrum (AdAS Spectrum), Autism Quotient questionnaire (AQ). Suicide risk was investigated by some specific items of the Trauma and Loss Spectrum-Self Report (TALS-SR) and putative PIU was identified on the basis of a specific AdAS Spectrum item. *Results*: 27.5% subjects reporting putative PIU. This subgroup showed higher scores in all domains of AdAS Spectrum and AQ compared with others. Students with putative PIU showed a significant correlation between suicide risk and the non-verbal communication domain of the AdAS Spectrum and the *Social skills* domain of the AQ. *Conclusions*: We found that students with PIU show higher levels of autistic traits compared to those without PIU. A significant correlation was found between autistic traits and suicide risk.

## 1. Introduction

Over the past decades, Internet users have steadily increased worldwide to almost 4.2 billion [1] and this progressive growth has led to an increasing focus on its related possible pathological consequences. The term problematic Internet use (PIU) was introduced almost two decades ago to describe a condition characterized by “use of the Internet that creates psychological, social, school and/or work difficulties in a person’s life” [2]. Since then, given the lack of a globally accepted definition or official diagnostic criteria in the current international nosography, Internet-related psychopathology has been variously named as PIU, Internet addiction, compulsive Internet use, pathological Internet use, and Internet dependence and has been widely studied in the literature [3,4,5,6,7,8,9]. Most recently, the World Health Organization (WHO) encoded the umbrella term ‘problematic use of Internet’ (PUI), encompassing all problematic Internet-related behaviors [10,11]. The core features of these conditions appear to be similar to those traditionally related to addictive disorders (such as loss of control, tolerance, withdrawal, and craving) and are often associated with intrapersonal and interpersonal conflicts and neglect in work, school, or family life [9,12]. Most recent literature has pointed out high comorbidity rates between PIU and neurodevelopmental disorders, like attention deficit hyperactivity disorder (ADHD) and, particularly, autism spectrum disorder (ASD), both in children and young adults [13,14,15,16]. Children with ASD showed significantly greater levels of problematic use of video games with respect to typically developing children, and this was true for both genders [17]. A cross-sectional study conducted by So et al. [14] explored the prevalence of Internet addiction among 132 adolescents with ASD and/or ADHD in a Japanese psychiatric clinic. The study showed rates of 10.8% and 20.0% in subjects with ASD alone and with comorbid ASD and ADHD, respectively.

MacMullin et al. [18] reported children and young adults with ASD to be atsignificantly higher risk for PIU compared with typically developing individuals. Furthermore, among adolescents with ASD the severity of PIU was higher than intypically developed peers. In a recent study, Engelhardt et al. [15] suggested higher risk for ‘problematic videogame use’in subjects with ASD, and this was especially related to some aspects of the behavioral phenotype of ASD, such as highly restricted interests, preoccupations, and repetitive behavior patterns [19]. For some individuals, these behavioral features may manifest as preoccupations with videogames or compulsive patterns of gameplay. However, conflicting results have also been reported. A study from Shane-Simpson et al. [20] involving 66 college students, in fact, failed to find a significant difference in levels of compulsive Internet usebetween subjects with ASD and normally developed ones.

The scant data reported so far on PIU and ASD in adult populations have highlighted an interesting association between autistic traits and PIU [16,21,22] in the framework of the increasing literature focusing on autistic traitsas risk factors for mental disorders and on their role in shaping psychopathological manifestations [23,24,25,26]. In a study by Finkenauer et al. [22], autistic traits were related with compulsive Internet useamong 195 married couples, particularly predicting increased compulsive Internet use overtime among women. Romano et al. [23] reported that Internet addiction was related to autistic traits in their sample. De Vries et al. [16], in a study on 231 Japanese psychiatric patients, found higher autistictraits among subjects with PIU with respect to normal Internet users.

A growing number of papers report a correlation between pathological levels of Internet use and an increased suicide risk [27,28,29]. In the last decades, websites concerning suicide proliferated exponentially. In particular, everyone can easily come upon different types of websites such as pro-suicide sites (encouraging suicide), sites describing methods but not encouraging suicide, sites providing factual information about suicide, as well as sites against suicides, providing support and promoting prevention [30]. Websites with self-harm or suicide content may normalize and reinforce self-injuring behaviors and suicidal thought [31,32]. Exposure to both suicide (either cluster suicides/contagion of suicidal behavior or media influence) and nonfatal suicidal behaviors is indeed predictive of a higher suicide risk in adolescents [33]. Furthermore, online search for suicide information in young people is related to not only depressive and social anxiety symptoms and suicidal ideation, but also to significantly higher suicide rates [28,34]. Results from a Korean epidemiological survey showed an association between Internet addictionand suicidal plans even after controlling for psychiatric disorders and sociodemographic factors [34]. Another cross-sectional study on 11,365 European adolescents reported PIU to be strongly related to suicidal behaviors [35]. On the other hand, a large cross-cultural survey reported that people may also use the Internet in order to obtain help and support: while some participants reported a reduction in self-harm, a small number reported increased self-harm associated with the use of websites concerning suicide. Despite these results, the relationship between general Internet use, PIU, and suicidal behavior was unclear, because lower levels of Internet use appeared to have a protective role when compared with no internet use at all [36].

Considering the evidence exposed so far, the aim of the present study was to investigate putative PIU among young university students attending three superior graduate schools in Italy, paying attention to possible correlation(s) with subthreshold autism spectrum symptoms and suicidality. Recent studies pointed out high ASD traits among high-achieving university students [37], but no data was reported on possible correlations with PIU or suicide behaviors. For this reason, we explored a selected population of high-achieving students, as represented by those attending the three Italian universities of excellence“Scuola Superiore Sant’Anna”, the “Collegio Universitario di Merito”, and the “Scuola Superiore di Catania”.

## 2. Methods

### 2.1. Study Participants

The study sample included 178 subjects comprised of students who responded to a recruitment campaign conducted in three Universities of excellence in Italy for the validation of the Adult Autism Subthreshold Spectrum (AdAS Spectrum) [37]: 63 (35.4%) participants were enrolled at the “Scuola Superiore Sant’Anna” (Pisa), 47 (26.4%) at the “Collegio Universitario di Merito” (Pavia), and 68 (38.2%) at the “Scuola Superiore di Catania” (Catania). All three of the schools are included among the “Superior Graduate Schools” of Italy that offer advanced training and research through university-type courses after passing highly selective entry tests. Among the 178 students, 93 were males and 85 were females. Mean age of the students enrolled was 21.24 ± 1.85 years. No a priori inclusion or exclusion criteria were used.

The school councils approved all recruitment and assessment procedures. The study was carried out in accordance with the Declaration of Helsinki. The Ethics Committee of the AziendaOspedaliero-Universitaria of Pisa approved all recruitment and assessment procedures (Ethical Approval Code n. 551/2015). Eligible subjects provided written informed consent after receiving a complete description of the study and having the opportunity to ask questions. Subjects were not paid for their participation in accordance with Italian law for clinical studies.A psychiatric visit with specialists in psychiatry (LDO, CC) of the Psychiatric unit of the Department of Clinical and Experimental Medicine (University of Pisa, Pisa) was offered to individuals reporting severe psychopathology or suicidal ideation or to whoever requesting it.

### 2.2. Instruments and Assessments

Enrolled students were asked to complete three self-report questionnaires: the Autism-Spectrum Quotient (AQ) [38] and the AdAS Spectrum [37], in order to evaluate the autistic features of the sample; and the Trauma and Loss Spectrum-Self Report (TALS-SR) [39,40], to investigate suicidality in enrolled subjects.

The AdAS Spectrum is a questionnaire developed by a group of researchers from the University of Pisa, within the framework of the Spectrum Project, an international Italy–USA research network. The questionnaire includes 160 items exploring the wide spectrum of manifestation of autism organized into seven domains: *Childhood/adolescence* (I); *Verbal communication* (II); *Non-verbal communication* (III); *Empathy* (IV); *Inflexibility and Adherence to Routine* (V); *Restricted interests and rumination* (VI); *Hyper- and hyporeactivity to sensory input* (VII). Item responses are coded in a dichotomous way (yes/no) and domain scores are obtained by counting the number of positive answers. The AdAS Spectrum hasalready been successfully used with this purpose in clinical and non-clinical settings [37,41,42,43]. Along with the aim of the present study, we focused on item number 66 of the AdAS Spectrum, which explores PIU: “*Do you spend a lot of time playing videogames or surfing on the Internet, to the extent of forgetting to do routine tasks?*” In particular, participants endorsing AdAS Spectrum item number 66 were defined as having putative PIU.

The Autism-Spectrum Quotient (AQ) is a widely used questionnaire developed to provide a self-report measure of autistic traits for use with adults with normal IQ [38]. It comprises 50 questions, assessing five different areas: *Social skills*; *Attention switching*; *Attention to detail*; *Communication*; *Imagination*. Test–retest and inter-rater reliability of the AQ were good, and items comprising each of the five domains showed moderate to high alpha coefficients, indicating reasonable construct validity. 

The TALS-SR is a questionnaire developed for assessing post-traumatic stress spectrum symptoms [39,40]. It includes 116 items exploring the lifetime experience of a range of losses and/or traumatic events and lifetime symptoms, behaviors, and personal characteristics that might represent manifestations and/or risk factors for the development of a stress response syndrome. According to the aims of the present study, we particularly focused on four items of the domain VII (Maladaptive Coping) assessing suicidal behaviors: item number 101(*…wish you hadn’t survived?*), item number 102 (*…think about ending your life?*), item number 103 (*intentionally scratch, cut, burn or hurt yourself?*), and item number 104 (*…attempt suicide?*).

### 2.3. Statistical Analysis

A Chi-square test was computed in order to compare the frequency of endorsement of AdAS Spectrum item number 66 in males vs. females. A comparison in the endorsement rates of at least one suicidality TALS-SR item between subjects with putative PIU and subjects without it was also calculated using a Chi-square test. Student’s *t*-tests were calculated to explore differences in each domain and total scores of the AdAS Spectrum and AQ questionnaires were used to determine the existence of differences in subjects with putative PIU compared to those without. Spearman’s correlation coefficients were computed in the subgroup of students with putative PIU to investigate possible associations between the AdAS Spectrum and AQ domains and the endorsement of the suicidal behavior TALS-SR items.

The statistical analyses were carried out using SPSS version 23.0 (IBM Corp., Armonk, NY, USA).

## 3. Results

Among the 178 students enrolled, 49 (27.5%) subjects presented putative PIU (34 males and 15 females, *p* = 0.008). When comparing mean AdAS Spectrum scores in subjects with and without putative PIU, statistically significant differences emerged for all domain scores with the only exception of *Hyper-hyporeactivity to sensory input*. In particular, the putative PIU group showed statistically significantly higher scores. Significant differences also emerged in the AQ total score as well as with AQ *Social Skills* and *Attention switching* domains between students with putative PIU and those without (see Table 1 for details).

Eighteen (10.3%) students endorsed at least one suicide behavior TALS-SR item, with no differences for gender (8 males (8.8%), 10 females (11.9%), *p* = 0.620), or group assignment (5 with PIU (10.2%), 13 without putative PIU (10.1%), *p* = 0.537). 

In the putative PIU group, a significant correlation emerged between the endorsement of the suicide behavior TALS-SR items and the *Non-verbal communication* mean domain score of the AdAS Spectrum (*R* = 0.395; *p* = 0.034) (see Table 2) and the *Social skills* domain of the AQ (R = 0.313; *p* = 0.34) (see Table 3).

## 4. Comments

To the best of our knowledge, this is the first study showing a link between adult autism subthreshold spectrum symptoms and suicide risk among students with putative PIU enrolled in universities of excellence in Italy. It is important to notice that we found higher rates of PIU compared to those previously reported by other European studies and similar to those found in Eastern countries [44]. Previous studies among Italian adolescent and young adults in the last years reported lower rates ranging between 0.7% and 14% [44,45]. A possible interpretation could be related to the use of a single item (item number 66 of the AdAS Spectrum) that, by encoding gaming and Internet use as a single construct, may overestimate the problem. On the other hand, the specific sample represented by university students attending special high-achieving schools may include subjects with high levels of computer use. 

Our results showed that university students with PIU had significantly higher scores in most domains of both the AdAS Spectrum and the AQ questionnaires. A possible interpretation of these results could suggest that students who spend most of their time on surfing the Internet or playing videogames instead of carrying out duties or other activities may do so because of a subthreshold autism spectrum that makes them prone to avoidance of interpersonal contacts and to solitary activities. Previous studies reported similar findings, corroborating the hypothesis of a role for autistic traits in PIU [16,21,22,44]. Romano et al. [46] found a significant correlation between Internet addiction and autistic traits evaluated by the AQ questionnaire among 60 adult volunteers. These findings were confirmed by a larger study on ninety participants, showing also the moderation effect of high levels of anxiety on this relationship [22]. Another study on 195 married couples, conducted by Finkenauer et al. [21], showed that autistic traits are related to PIU both in women and men. These data suggest that due to their social inhibition and poor communication skills, subjects with autistic traits may choose online interactions. Communication via the Internet is in fact simpler in some ways and does not require non-verbal skills or social abilities. This preference for online interactions may, on the other hand, lead to a decrease of ’offline’ relationships and to an increased likelihood of developing PIU as well [47].

Furthermore, the results of the present study showed that in subjects with putative PIU, suicide risk was related to the AdAS Spectrum *Non-verbal communication* and to the AQ *Social skills* domains. Several studies show how patients with ASD present a higher risk of suicide with respect to the general population [48,49,50]. Suicide behavior rates among adults and young adults with ASD were found to range between 7.3% to 50%, with suicide ideation ranges from 31% to 50%, and incidence of suicide attempts of 7.7% [51]. Impairment in communication skills or the reduced reciprocity associated with ASD could lead to social isolation and self-reported loneliness [14,52,53]. Social isolation is a well-acknowledged risk factor for suicide in the general population [54] and in non-clinical samples [55]. Furthermore, a thwarted sense of belongingness was recently recognized as a mediator in the relationship between autistic traits and suicide [56]. In individuals where PIU could lead to relationship problems and loneliness, it is easy to imagine how an intrinsic impairment in communication abilities represent a relevant risk factor for social isolation and consequently to an increased risk ofsuicide. Concerning these dangerous relationships, Finkenauer and colleagues [21] recommended that Internet use should be carefully controlled in individuals with ASD to avoid an interference on their ‘offline’ contacts.

While interpreting the results of the present study, some important limitations should be taken into account. First, the limited sample size needs to be considered. However, it should be noted that it is quite large considering the strict selection requirements of the universities of excellence chosen for the study. Second, there was a lack of a specific scale assessing PIU. With that said, it should be noted that different definitions of this construct are still currently being reported in literature, so at present a widely recognized scale for assessing PIU is lacking. The use of a single AdAS Spectrum item may in fact impact on the estimates of this behavior. This item does not represent a specific instrument for assessing a complex condition like PIU, and for this reason, we addressed it as putative PIU. A bias may also be derived from the fact that it combines gaming and internet use, thus not allowing for a distinctive measure of these concepts. Another major limitation is represented by the use of self-report questionnaires to investigate subthreshold autistic traits and suicidality. A self-report instrument may in fact be less accurate than the rating of a clinician to evaluate symptomatology. However, most of the previous studies on PIU use AQ to assess levels of autistic traits in their samples [16,20,21,22,46]. The third limitation is the lack of information on mental disorders in the sample that could be associated with suicide.

## 5. Conclusions

The present study corroborates previous studies showing higher autistic traits in individuals with PIU. These data point out the relationship between autistic features and pathological levels of Internet use in a non-clinical population. Consequently, putative PIU should be evaluated by clinicians during the assessment of individuals with high autistic traits. Moreover, investigating ASD symptomatology may be useful when investigating patients with putative PIU. Furthermore, this is the first study to suggest how an impairment in social skills and in non-verbal communication, typical of autism symptomatology, may be correlated to suicide risk in individuals with putative PIU. Autism spectrum features like difficulties in social relationships and isolation might be a warning sign of increased suicide risk in subjects spending most of their time on the Internet. Future longitudinal studies are needed to better understand both the risk of PIU and the risk of suicide in young adults with high levels of autistic traits, also in relation to the medical-legal issues deriving from an erroneous diagnostic classification in relation to the new legislation in force.

## Figures and Tables

**Table 1:** Adult Autism Subthreshold Spectrum (AdAS Spectrum) and Autism Quotient questionnaire (AQ) scores in superior university students with putative problematic Internet use (PIU) (*n* = 49) with respect to those without (*n* = 129).

	no PIU	PIU	*p*
**AdAS Spectrum**			
(I) Childhood/adolescence	6.4 (±18.9)	8.8 (±17.3)	<0.001
(II) Verbal communication	4.6 (±16.5)	5.7 (±14.5)	0.023
(III) Non-verbal communication	8.8 (±14.9)	13.4 (±16.5)	<0.001
(IV) Empathy	2.3 (±17.7)	3.3 (±19.3)	0.014
(V) Inflexibility and adherence to routine	12.6 (±15.5)	15.3 (±13.9)	0.017
(VI) Restricted interests and rumination	7.5 (±20.2)	10 (±14.8)	<0.001
(VII) Hyper- and hyporeactivity to sensory input	3.4 (±16.1)	4.3 (±16.8)	0.051
AdASSpectrumtotal score	46 (±21.2)	61.2 (±18.2)	<0.001
**AQ**			
Social skills	2.6(±2.5)	3.5 (±2.3)	0.026
Attention switching	4.2 (±2.0)	5.1 (±2.2)	0.014
Attention to detail	4.6 (±2.3)	5.1 (±1.9)	0.144
Communication	2.4 (±2.0)	2.7 (±1.9)	0.500
Imagination	2.8 (±1.6)	2.8 (±1.7)	0.986
Total AQ	16.7 (±6.7)	19.5 (±6.2)	0.013

**Table 2:** Correlation between the AdAS Spectrum domains and total score and the Trauma and Loss Spectrum-Self Report (TALS-SR) suicide behavior score in superior university students with putative PIU.

AdAS Spectrum	Suicide Behavior Score (*R*, *p*)
(I)Childhood/adolescence	−0.013, 0.930
(II)Verbal communication	0.062, 0.680
(III) Non-verbal communication	0.395, 0.007
(IV) Empathy	−0.010, 0.947
(V) Inflexibility and adherence to routine	0.143, 0.345
(VI) Restricted interests and rumination	0.165, 0.274
(VII) Hyper- and hyporeactivity to sensory input	0.279, 0.060
AdAS Spectrum total score	0.207, 0.167

**Table 3:** Correlation between the AQ domains and total score the TALS-SR suicide behavior score in superior university students with putative PIU.

AQ	Suicide Behavior Score (*R*, *p*)
Social skills	0.313, 0.034
Attention switching	0.055, 0.716
Attention to detail	0.218, 0.146
Communication	0.177, 0.245
Imagination	0.150, 0.327
AQ total score	−0.188, 0.222
